# Feasibility, uptake, and exploratory outcomes of an individualized exercise program in pediatric oncology: a prospective single-center pilot study

**DOI:** 10.3389/fped.2026.1914065

**Published:** 2026-08-13

**Authors:** Christina Schuster, Elena Loos, Thomas Traunwieser, Michael C. Frühwald, Michaela Kuhlen

**Affiliations:** Pediatrics and Adolescent Medicine, Faculty of Medicine, University of Augsburg, Augsburg, Germany

**Keywords:** exercise therapy, feasibility, pediatric oncology, physical activity, quality of life, supportive care

## Abstract

**Introduction:**

Exercise interventions may help mitigate treatment-related physical impairment in pediatric oncology, but implementation in routine care remains challenging, particularly during active treatment and in heterogeneous patient cohorts.

**Patients:**

This prospective single-center pilot study evaluated an individualized exercise program for children and adolescents aged 4–17 years undergoing cancer treatment during the COVID-19 pandemic.

**Method:**

The program combined supervised in-hospital exercise, personalized home-based training, and an optional video-based SMART-Sport component introduced from month 3. The primary focus was feasibility, uptake, safety, and acceptability. Motor performance, six-minute walk distance, health-related quality of life (HRQoL), and neuropsychological functioning were assessed at baseline and six months as secondary exploratory outcomes.

**Results:**

Twenty participants were enrolled; 15 completed at least one six-month follow-up assessment. Overall, 348 of 583 scheduled in-hospital exercise opportunities were attended, corresponding to a scheduled-session attendance of 59.7% using a conservative denominator. No serious program-related adverse event occurred. Movement diaries were available for 15 participants and documented 361 home-training sessions, of which 262 (72.6%) occurred during the first three months. Four participants documented use of SMART-Sport, completing 14 sessions. Exploratory complete-case analyses showed nominal within-participant improvements in hand-eye coordination, reaction time, six-minute walk distance, and selected HRQoL domains. Neuropsychological findings were inconclusive.

**Discussion:**

The findings support the feasibility and safety of supervised individualized in-hospital exercise under the conditions of a specialized pediatric oncology center with a dedicated sports therapist. Feasibility was component-specific: home-training documentation decreased over time, and uptake of the optional video-based component was limited.

**Conclusion:**

This pilot study provides preliminary implementation data supporting further development of individualized exercise programs in pediatric oncology. Exploratory outcome findings should be interpreted as hypothesis-generating and require confirmation in larger controlled studies.

## 1 Introduction

A cancer diagnosis during childhood or adolescence can substantially disrupt physical, psychological, and social development (1). Intensive multimodal treatment, including chemotherapy, radiotherapy, and surgery, is frequently accompanied by reduced physical activity, muscle weakness, impaired motor performance, and reduced cardiorespiratory fitness (1–7). These impairments may persist beyond the end of treatment and contribute to long-term reductions in health-related quality of life (HRQoL) and an increased risk of secondary health complications (5, 8–11).

A growing body of evidence supports the role of structured exercise as a component of supportive care in pediatric oncology. Exercise interventions have generally been shown to be safe and feasible during both acute cancer treatment and follow-up care (12–17). Reported potential benefits include improvements in fatigue, muscular strength, cardiorespiratory fitness, motor performance, physical functioning, and HRQoL (12, 18–25). Physical activity may also be relevant to neuropsychological functioning including attention, executive functioning, attention, and working memory, although evidence during active pediatric cancer treatment remains limited (26–29).

Despite increasing evidence and corresponding clinical recommendations, structured exercise programs remain inconsistently implemented in routine pediatric oncology care. Reported barriers include concerns regarding patient safety, limited personnel and institutional resources, and the need to accommodate substantial differences in age, diagnosis, treatment phase, symptoms, and daily physical condition (30, 31). These challenges are particularly relevant during active treatment, when medical appointments, acute toxicity, hospitalization patterns, and infection-control measures may interfere with regular participation.

During the COVID-19 pandemic, we implemented an individualized multimodal exercise program at the Swabian Children's Cancer Center. The program combined supervised in-hospital exercise with personalized home-based training. From the third month of participation, an optional video-based exercise component, SMART-Sport, was added for participants considered suitable for remote training.

The primary aim of this prospective single-center pilot study was to evaluate the feasibility, uptake, safety, and acceptability of this individualized exercise program during pediatric cancer treatment. Feasibility and implementation-related outcomes were therefore the main focus of the study. Changes in motor performance, endurance, HRQoL, and neuropsychological functioning over six months were analyzed as secondary exploratory outcomes to describe potential outcome signals and inform future controlled studies, not to establish intervention efficacy. Uptake of the optional SMART-Sport component was evaluated separately and descriptively.

## 2 Patients and methods

### 2.1 Study design and participants

We conducted a prospective, single-center, non-randomized pilot study at the Swabian Children's Cancer Center, University Hospital Augsburg, Germany. Participants were recruited between May 2020 to April 2021 during the COVID-19 pandemic and were followed individually for six months after enrollment. Participants entered the study at different stages of cancer treatment according to their treatment status at the beginning of the recruitment period or at the time of subsequent enrollment.

Children and adolescents aged 4–17 years with an oncological diagnosis who were receiving treatment at the center were eligible. Exclusion criteria were:

- Age below four years;
- Insufficient understanding of German or English;
- Intellectual disability preventing meaningful participation in the exercise program.
- and a permanent medical contraindication to endurance interval exercise and/or resistance training, such as impaired myocardial function;
- Temporary medical limitations affecting individual exercise sessions did not necessarily result in exclusion from the study.

No formal sample-size calculation was performed. The sample size was determined pragmatically by the number of eligible participants recruited during the predefined recruitment period. Accordingly, the study was not powered to detect intervention effects or to support subgroup analyses.

The study was approved by the Ethics Committee of LMU Munich (20-073) as a prospective single-center feasibility study assessing the implementation and uptake of an age-appropriate exercise program in pediatric oncology care. Written informed consent was obtained from the participantś legal guardians, and age-appropriate assent was obtained from participating children and adolescents. The study was conducted in accordance with the Declaration of Helsinki.

The study was not registered in a public clinical trials registry before enrollment. This limits methodological transparency and is acknowledged as a limitation. The study was not designed as a confirmatory efficacy trial and did not include randomization, a control group, or allocation to alternative treatment strategies.

Assessments were originally scheduled at baseline, three months, and six months. For the present report, analyses were restricted to baseline and six-month data because the six-month assessment represented the predefined endpoint of the feasibility observation period and provided the most complete longitudinal assessment of the intervention period. The three-month assessment of the intervention was considered an interim time point and was not included in the present analysis in order to avoid additional outcome multiplicity and to maintain a focused comparison between baseline and completion of the six-month program.

### 2.2 Study outcomes

The primary outcomes of this feasibility study were implementation-related and included delivery of the exercise program, attendance at scheduled in-hospital exercise opportunities, participation in home-based training, uptake of SMART-Sport, reasons for non-attendance, adverse events, and participant and caregiver feedback. Motor performance, endurance, HRQoL, and neuropsychological functioning were assessed as secondary exploratory outcomes to describe potential changes over time and to generate hypotheses for future controlled studies, not to provide confirmatory evidence of efficacy.

### 2.3 Exercise program

The individualized exercise program comprised supervised in-hospital exercise, personalized home-based training, and an optional video-based component introduced during the second half of the six-month observation period. Exercise content was adapted to the participant's age, diagnosis, treatment status, symptoms, functional capacity, motivation, and personal preferences. One sports therapist (CS) delivered and supervised the exercise program.

Although the program was individualized, it followed a structured framework to support consistency across participants. Each supervised session was planned within predefined exercise domains, including mobilization, strength, endurance, coordination, balance, flexibility, and relaxation. The selection and weighting of these components depended on the participant's age, diagnosis, treatment phase, current symptoms, functional capacity, and motivation.

Exercise intensity was adapted at each session according to the participant's daily medical condition, observed fatigue, symptoms, and perceived exertion. Where age and understanding permitted, perceived exertion was guided by the Borg Rating of Perceived Exertion scale, with moderate intensity generally targeted. Progression was applied individually when previous sessions had been tolerated well and no new medical limitations were present. Depending on the exercise type, progression included increasing duration, repetitions, number of sets, external resistance, walking or cycling time, balance difficulty, or coordinative complexity. Conversely, exercise volume, intensity, or complexity was reduced when participants had treatment-related symptoms, fatigue, pain, reduced general condition, or limited motivation.

Consistency was supported by delivery through a single sports therapist, use of predefined institutional safety criteria before each session, a common session structure where clinically appropriate, and prospective documentation of scheduled sessions, attended sessions, session duration, exercise content, reasons for non-attendance, and adverse events.

#### 2.3.1 Supervised in-hospital exercise

In-hospital exercise sessions were scheduled primarily on weekdays for both inpatients and participants attending the day clinic. A small number of Saturday sessions was also offered when feasible. Before each session, the sports therapist assessed whether exercise was appropriate using predefined institutional criteria for exercise during pediatric cancer treatment. In cases of uncertainty, suitability was discussed with the treating physician.

Sessions were individualized according to the participant's current medical condition, physical capacity, age, motivation, and available time. Session duration ranged from approximately 10 to 95 min. Sessions generally included an age-appropriate warm-up or active mobilization, an individualized main exercise component, and a cool-down or relaxation phase. In brief sessions or when participants were physically limited, individual components were shortened or omitted as clinically appropriate.

Strength training included functional body-weight exercises and exercises using resistance bands, dumbbells, medicine balls, or vibration plates. Endurance activities included stationary cycling, walking in the ward corridor, and movement-based games. Coordination training included balance exercises, reaction tasks using visual or auditory stimuli, and combined motor-cognitive activities, such as balance exercises accompanied by memory tasks or simple arithmetic.

#### 2.3.2 Personalized home-based training

Participants received individualized recommendations for exercise at home. Training plans were adapted to age, medical and functional status, interests, and the equipment, games, or everyday objects available in the household. The plans included activities targeting strength, endurance, flexibility, and coordination. Suggested endurance activities included walking, cycling, playground activities, and active movement or running games. Coordination activities included balance exercises using household objects and movement tasks that could be performed together with parents or other family members.

Participants were generally advised to exercise two to three times per week for approximately 20–30 min. Perceived exertion was monitored using the Borg Rating of Perceived Exertion scale where age and understanding permitted its use. A perceived exertion level of 11–14 on the 6–20 scale was recommended.

Home exercise was recorded in a movement diary by the participant, a caregiver, or both. Diaries were reviewed regularly by the sports therapist, who provided additional guidance where necessary. Home-based training was intended to continue throughout the six-month observation period in parallel with in-hospital exercise whenever participants were at home and medically able to exercise. Because hospitalization patterns varied between participants, home-based training opportunities were not standardized by calendar week or treatment phase.

Movement-diary data were not available for all enrolled participants because not every participant received or returned a diary. Home-training analyses were therefore restricted to participants with available diary data. Missing diary data were treated as missing and were not interpreted as absence of home exercise. The movement diary documented completed home-training sessions but did not systematically capture all recommended but not-completed sessions or reasons for non-completion. Therefore, home-training data were interpreted as documentation of recorded participation rather than as a complete measure of adherence to the recommended home-exercise frequency.

#### 2.3.3 SMART-sport

SMART-Sport was an optional video-based exercise component introduced approximately three months after enrollment. It was not intended as a mandatory intervention component or as a replacement for supervised in-hospital exercise, but as an additional home-based video component for participants considered suitable for remote exercise. Suitability was assessed by the sports therapist based on age, general condition, previous exercise experience, ability to understand and follow the video instructions, need for caregiver support, and expected ability to perform the exercises safely in the home environment.

The exact number of participants to whom SMART-Sport was offered was not systematically documented and could not be reconstructed retrospectively. Therefore, uptake was reported descriptively as the number of participants who documented at least one completed SMART-Sport session among the total enrolled cohort.

The approximately 30 min videos were tailored to three age groups: 5–8 years, 9–13 years, and 14–17 years. Each video included an age-appropriate warm-up, a main component comprising coordination and strengthening exercises, and a cool-down or relaxation phase. The warm-up consisted of active mobilization, movement games, or age-appropriate movement stories. The main component included a coordination task, such as single-leg stance, and two or three exercises targeting the lower limbs, core, and upper limbs. Exercises included squats, wall sits, bridging, and planks. Easier alternatives were provided to allow adaptation to the participant's current physical capacity and to reduce the risk of overexertion. The cool-down included stretching or relaxation exercises.

Participants and caregivers received safety instructions before using the videos. They were advised to complete the videos once or twice per week and to document completed sessions in their movement diary. For the present analysis, SMART-Sport sessions completed between its introduction at approximately month 3 and the six-month assessment were included. The maximum observation period for SMART-Sport was therefore approximately three months. Because SMART-Sport was optional, introduced later than the other program components, and not offered to every participant, its uptake was analyzed separately and descriptively.

### 2.4 Assessments

Assessments were performed at baseline (T0) and at the six-month follow-up (T1). Where an assessment could not be completed on one day because of treatment procedures, fatigue, reduced general condition, or logistical constraints, testing was divided across more than one day where clinically appropriate.

The Motor Performance in Pediatric Oncology test battery (MOON test) and six-minute walk test (6MWT) were administered by the sports therapist. The PedsQL questionnaire was administered by either the sports therapist or a neuropsychologist (EL). Neuropsychological assessments were conducted by a neuropsychologist (EL).

#### 2.4.1 Demographic and medical data

Demographic and medical information was obtained from the medical records at baseline and updated during the study where necessary. Data included age at study enrollment, sex, oncological diagnosis, treatment modality, treatment status, relevant comorbidities, and clinical contraindications to exercise.

#### 2.4.2 Motor performance

Motor performance was assessed using the MOON test (32, 33). The analyzed tasks assessed hand-eye coordination, static balance, reaction time, flexibility, explosive strength, sit-to-stand performance, and handgrip strength.

Outcomes were reported in their original measurement units: hand-eye coordination, reaction time, and sit-to-stand performance in seconds; static balance as the number of contacts; flexibility in centimeters; explosive strength in meters; and handgrip strength in kilograms.

The raw MOON tasks were considered suitable for longitudinal within-participant comparisons in children aged four and five years. However, because normative MOON reference values were unavailable for this age group, participants aged four and five years were excluded from analyses involving comparisons with normative values but were retained in analyses of raw paired measurements.

Functional endurance was assessed using the standardized 6MWT (34, 35). Participants were instructed to cover as much distance as possible during six minutes according to the standardized test procedure. The outcome was the total distance walked, reported in meters.

#### 2.4.3 Health-related quality of life

HRQoL was assessed using the German age-appropriate versions of the PedsQL 4.0 Generic Core Scales (36–38). The physical, emotional, and social functioning domains were included in the present analysis.

Age-appropriate child self-report versions were used from the age of five years. Participants aged four years contributed parent proxy-report data only. Parent proxy-report versions were completed by a caregiver. Items were reverse-scored and linearly transformed to a scale ranging from 0 to 100 in accordance with the standardized scoring procedure. Higher scores indicated better HRQoL.

#### 2.4.4 Neuropsychological functioning

Cognitive functioning was assessed using a study-specific battery of standardized, age-appropriate neuropsychological tests. The battery was designed to assess fluid reasoning, verbal short-term memory, verbal and visual working memory, processing speed, attention, inhibitory control, and cognitive flexibility (39–41).

Fluid reasoning was assessed using Raven's Progressive Matrices. Verbal short-term memory was assessed using age-appropriate forward digit-span tasks from the Kaufman Assessment Battery for Children, Second Edition, the Wechsler Intelligence Scale for Children, Fifth Edition, or the Wechsler Adult Intelligence Scale, Fourth Edition. Verbal working memory was assessed using backward digit-span tasks from the Wechsler Intelligence Scale for Children, Fifth Edition, or the Wechsler Adult Intelligence Scale, Fourth Edition. Visual working memory was assessed using the Picture Memory subtest of the Wechsler Preschool and Primary Scale of Intelligence, Fourth Edition, or the Picture Span subtest of the Wechsler Intelligence Scale for Children, Fifth Edition. Processing speed was assessed using age-appropriate subtests from the Wechsler Preschool and Primary Scale of Intelligence, Fourth Edition, the Wechsler Intelligence Scale for Children, Fifth Edition, or the Wechsler Adult Intelligence Scale, Fourth Edition. These included Bug Search or Animal Coding, Coding, and Symbol Search. Attention, inhibitory control, and cognitive flexibility were assessed using the Alertness, Go/No-Go, and Incompatibility subtests of the Test of Attentional Performance.

Tests were administered and scored according to the respective test manuals. To permit results from different age-appropriate instruments to be summarized across participants, age-standardized T-scores were used. T-scores have a normative mean of 50 and a standard deviation of 10. Higher values in the reported variables indicated better age-adjusted performance.

Because the number of paired observations was very small for several neuropsychological domains, particularly the attentional measures, these outcomes were considered exploratory and were primarily summarized descriptively.

### 2.5 Feasibility, safety, and acceptability

Feasibility was evaluated descriptively across four implementation domains: program delivery, uptake, safety, and acceptability. Program delivery was assessed by whether supervised in-hospital sessions, home-based recommendations, and the optional SMART-Sport component could be offered within routine care. Uptake was assessed using scheduled-session attendance, documented home-training sessions, SMART-Sport use, and reasons for non-attendance. Safety was assessed by documentation of serious and minor adverse events occurring in temporal association with exercise. Acceptability was assessed using participant and caregiver feedback at the six-month follow-up.

No predefined numerical feasibility threshold or formal success criterion was specified in the study protocol. Therefore, feasibility was not judged against a formal success benchmark but interpreted pragmatically based on the combined pattern of implementation indicators.

Scheduled-session attendance was calculated as attended supervised in-hospital sessions divided by all scheduled exercise opportunities during the six-month observation period. Scheduled opportunities were retained in the denominator irrespective of hospital presence or medical suitability on the respective day. The reported rate therefore represents a conservative real-world implementation measure and may underestimate participation among participants who were physically present and medically able to exercise. Because participant presence and day-to-day medical suitability were not documented independently for all scheduled opportunities, a more restrictive attendance rate could not be calculated reliably.

Home-training participation was summarized only for participants with available movement-diary data. SMART-Sport uptake was evaluated using sessions documented between month 3 and the six-month assessment.

Participant and caregiver experiences were assessed at the six-month follow-up using a study-specific questionnaire. Participants were asked, among other items, whether they enjoyed the exercise program and whether exercise provided a welcome distraction from their illness. Caregivers were asked whether they perceived an improvement in their child's mood following exercise.

### 2.6 Statistical analysis

Statistical analyses were performed using IBM SPSS Statistics, version 29.0.1.0. Descriptive statistics were used to summarize participant characteristics, feasibility outcomes, exercise participation, adverse events, and participant feedback. Continuous variables were reported as means and standard deviations. Given the small sample size and potentially skewed distributions, medians and ranges or interquartile ranges were additionally reported where informative. Categorical variables were presented as frequencies and percentages.

Missing data were not imputed. Feasibility outcomes were summarized using all enrolled participants for whom the respective data source was available. Home-training analyses were restricted to participants with available movement-diary data. Exploratory motor-performance, endurance, HRQoL, and neuropsychological analyses were performed using complete paired observations for each respective outcome. Therefore, the analysis population differed between outcomes, and the paired sample size is reported separately in each table. Because of the small cohort size, no formal comparison between participants with and without complete follow-up data was performed.

Baseline and six-month outcomes were compared using participants with paired observations for the respective outcome. The distribution of within-participant change scores was assessed using graphical inspection and the Shapiro–Wilk test. Approximately normally distributed differences were analyzed using two-sided paired t-tests. Non-normally distributed differences were analyzed using two-sided Wilcoxon signed-rank tests. Effect sizes were reported as Cohen's dz for paired t-tests and matched-pairs rank-biserial correlations for Wilcoxon signed-rank tests. Where estimable, 95% confidence intervals were reported for changes and effect estimates.

All statistical tests were two-sided, and nominal *p* values < 0.05 was reported. Because this was an exploratory pilot study involving multiple outcomes in a small and heterogeneous cohort, no adjustment for multiple comparisons was performed. The resulting *p*-values should therefore be interpreted descriptively and as hypothesis-generating only, not as confirmatory evidence of intervention efficacy. To reduce the risk of overinterpretation, emphasis was placed on paired sample sizes, direction and magnitude of within-participant changes, and consistency with the feasibility and implementation findings rather than on statistical significance alone.

Outcomes with fewer than five paired observations were summarized descriptively without formal significance testing.

## 3 Results

### 3.1 Participant flow and cohort characteristics

**Figure 1:**
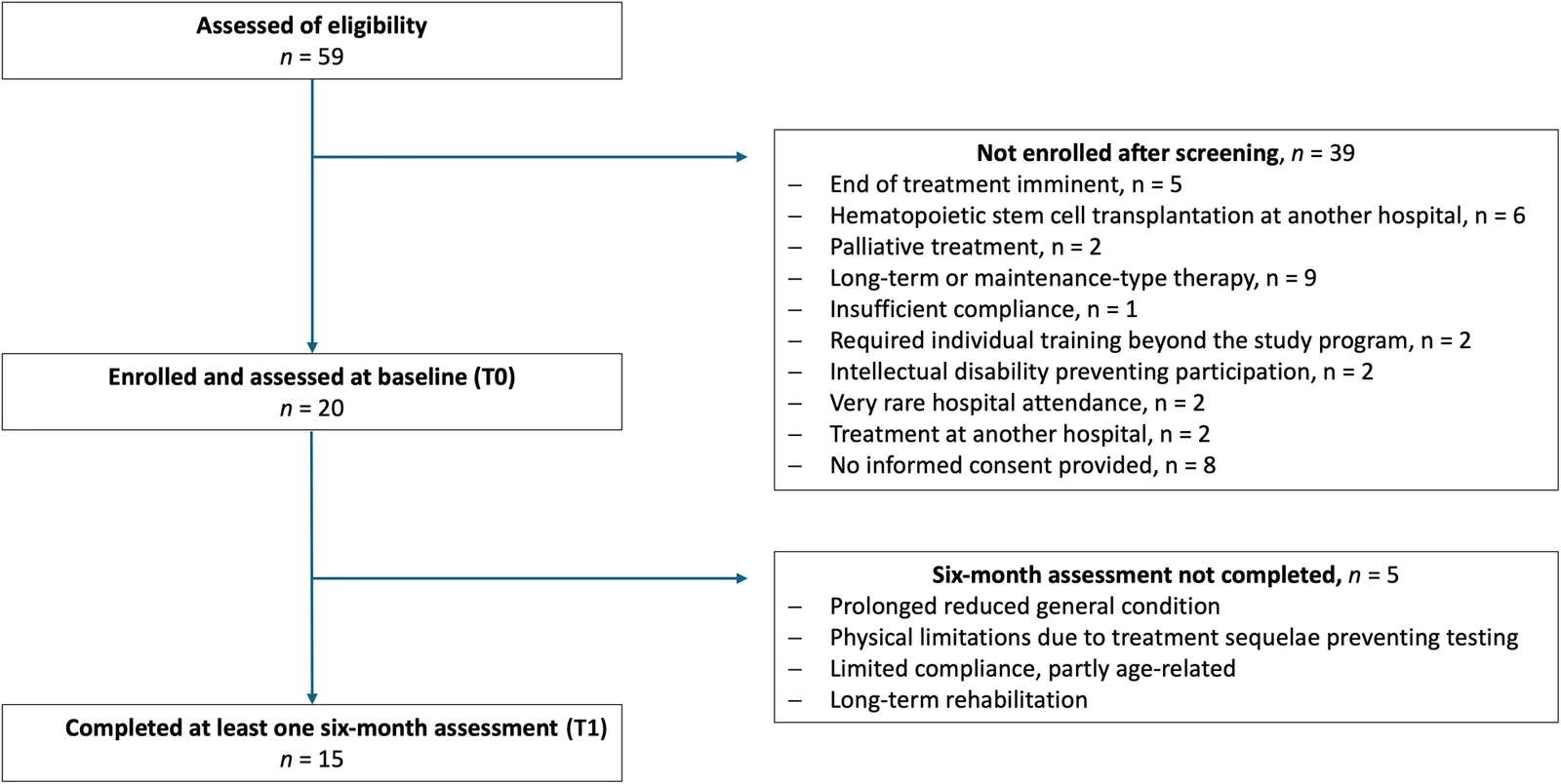
Participant flow through screening, enrollment, and follow-up. T0 denotes baseline and T1 denotes the six-month assessment.

During the recruitment period, 59 children and adolescents were assessed for eligibility. Of these, 39 were not enrolled after screening. Reasons are shown in Figure 1. Twenty participants were enrolled and completed the baseline assessment.

At least one assessment at the six-month follow-up was completed by 15 of the 20 enrolled participants. Five participants did not complete the six-month assessment. Reasons included prolonged reduced general condition, physical limitations due to treatment sequelae that prevented testing, limited compliance partly related to young age, and participation in long-term rehabilitation.

The enrolled cohort included 11 females and 9 males. The median age at enrollment was 7.0 years, with a range of 4 to 17 years. Half of the cohort had acute lymphoblastic leukemia (*n* = 10, 50%). The remaining diagnoses comprised lymphoma (*n* = 4, 20%), central nervous system tumors (*n* = 2, 10%), sarcomas or other solid tumors (*n* = 2, 10%), acute myeloid leukemia (*n* = 1, 5%), and neuroblastoma (*n* = 1, 5%). Participants entered the study at different stages of cancer treatment. Demographic and clinical characteristics of the cohort are summarized in Table 1.

**Table 1:** Demographic and clinical characteristics of the study cohort.

Characteristic	Overall cohort (*N* = 20)
Age at enrollment, years	
Mean ± SD	8.2 ± 4.1
Median (range)	7.0 (4–17)
Age group, *n* (%)	
4–5 years	8 (40)
6–11 years	7 (35)
12–17 years	5 (25)
Sex, *n* (%)	
Female	11 (55)
Male	9 (45)
Oncological diagnosis, *n* (%)	
Acute lymphoblastic leukemia	10 (50)
Acute myeloid leukemia	1 (5)
Hodgkin lymphoma	2 (10)
Non-Hodgkin lymphoma	1 (5)
Burkitt lymphoma	1 (5)
Central nervous system tumors	2 (10)
Neuroblastoma	1 (5)
Ewing sarcoma	1 (5)
Soft-tissue tumor	1 (5)
Treatment modality, *n* (%)	
Chemotherapy only	12 (60)
Chemotherapy and radiotherapy	2 (10)
Chemotherapy and surgery	3 (15)
Radiotherapy and surgery	1 (5)
Chemotherapy, radiotherapy, and surgery	2 (10)

Age was calculated in completed years at the date of study enrollment. Data are presented as *n* (%) unless otherwise indicated. SD, standard deviation.

The availability of paired follow-up data differed between outcome domains. Paired observations were available for 9 to 15 participants for motor-performance and endurance outcomes, for 10 participants for child-reported HRQoL, for 11 to 14 participants for parent proxy-reported HRQoL, and for 7 to 11 participants for the main neuropsychological domains. Attentional subtests were therefore summarized descriptively without formal statistical testing. Movement-diary data for home-based training were available for 15 participants.

### 3.2 Feasibility and exercise participation

During the six-month observation period, 583 supervised in-hospital exercise opportunities were scheduled for the 20 enrolled participants. A total of 348 sessions were attended, corresponding to an overall scheduled-session attendance of 59.7% using the predefined conservative denominator. (Table 2)

**Table 2:** Delivery and uptake of the individualized exercise program during the six-month observation period.

Component and Measure	Participants or Observations contributing, n	Total	Mean ± SD	Median (range)
Supervised in-hospital exercise				
Scheduled exercise opportunities per participant	20	583	29.2 ± 17.0	28 (1–73)
Attended sessions per participant	20	348	17.4 ± 11.7	16 (1–43)
Scheduled-session attendance, %	20	348/583 (59.7%)	60.1 ± 22.6	60.4 (12.5–100)
Duration of attended sessions, min	348 sessions	-	33.8 ± 9.7	30 (10–95)
Home-based training				
Participants with available movement-diary data	15	15/20 (75.0%)	-	-
Sessions during months 0–3	15	262	17.5 ± 12.2	15 (0–36)
Sessions during months 4–6	15	99	6.6 ± 11.5	3 (0–45)
Sessions during the complete six-month period	15	361	24.1 ± 21.5	17 (0–80)
SMART-Sport				
Participants documenting at least one session	4	4/20 (20.0%)	-	-
SMART-Sport sessions per user	4	14	3.5 ± 1.3	3.5 (2–5)

Scheduled-session attendance was calculated for each participant as attended in-hospital sessions divided by all scheduled exercise opportunities during the six-month observation period. Scheduled opportunities were retained in the denominator irrespective of hospital presence or medical suitability on the respective day. The reported rate therefore represents a conservative real-world implementation measure and may underestimate participation among participants wo where physically present and medically able to exercise. Home-training analyses included only participants with available movement-diary data. Missing diaries were not interpreted as zero exercise. SD, standard deviation.

At the participant level, the mean scheduled-session attendance was 60.1 ± 22.6%, with a median of 60.4% and a range of 12.5%–100%. Participants attended a mean of 17.4 ± 11.7 supervised sessions, with a median of 16 sessions and a range of 1–43. The mean duration of attended sessions was 33.8 ± 9.7 min; the median duration was 30 min, with a range of 10–95 min.

Of the 235 scheduled opportunities that were not attended, reduced general condition or other symptoms accounted for 54 missed sessions (23.0%), logistical unavailability for 49 (20.9%), concurrent treatment or diagnostic procedures for 42 (17.9%), refusal or lack of motivation for 36 (15.3%), infection-control measures including COVID-19-related isolation or quarantine for 12 (5.1%), and other reasons for 2 (0.9%). No reason was documented for 40 missed opportunities (17.0%).

No serious adverse event was attributed to the exercise program. Minor events occurring in temporal association with exercise included transient nausea, one fall without injury, temporary exacerbation of pain, and exercise-related muscle soreness.

### 3.3 Home-Based training

Movement-diary data were available for 15 of the 20 enrolled participants. These participants documented a total of 361 home-training sessions between baseline and the six-month assessment (Table 2). Participants with available diaries completed a mean of 24.1 ± 21.5 home-training sessions, with a median of 17 sessions and a range of 0–80. One participant with an available diary recorded no home-training sessions.

Most documented home exercise occurred during the first half of the observation period. A total of 262 sessions, corresponding to 72.6% of all documented home-training sessions, were completed during months 0–3. The remaining 99 sessions, or 27.4%, were completed during months 4–6. Because the diaries recorded completed sessions but did not systematically document reasons for non-completion, the causes of this decrease could not be determined at the individual level.

### 3.4 Uptake of SMART-Sport

Four participants documented use of the optional SMART-Sport videos between approximately month 3 and the six-month assessment. Together, they completed 14 SMART-Sport sessions. Among participants who used SMART-Sport, the mean number of completed sessions was 3.5 ± 1.3, with a median of 3.5 sessions and a range of 2–5.

Because the number of participants to whom SMART-Sport was offered was not systematically documented and could not be reconstructed retrospectively, the uptake rate among eligible or offered participants could not be calculated.

### 3.5 Motor performance and endurance

Paired motor-performance and endurance data were available for 9 to 15 participants, depending on the individual test item. The exact paired sample size is reported for each outcome in Table 3.

**Table 3:** Changes in motor performance and endurance between baseline and the six-month assessment.

Outcome	Paired observations, n	Baseline T0, mean ± SD	Six months T1, mean ± SD	Mean change	Test	Nominal two-sided *p*-value	Effect size
Hand-eye coordination, sec	15	67.5 ± 20.8	56.7 ± 13.7	−10.8	Wilcoxon	0.002	−0.90
Static balance, contacts	13	18.8 ± 8.9	15.4 ± 6.9	−3.4	Paired *t*-test	0.172	−0.40
Reaction time, sec	15	0.372 ± 0.105	0.332 ± 0.081	−0.040	Wilcoxon	0.002	−0.85
Flexibility, cm	13	−10.1 ± 8.3	−8.9 ± 8.2	1.2	Wilcoxon	0.280	0.37
Explosive strength, m	9	3.4 ± 0.7	3.4 ± 0.8	0.1	Paired *t*-test	0.559	0.20
Sit-to-stand, sec	14	9.4 ± 4.0	8.4 ± 1.5	−1.1	Wilcoxon	0.296	−0.33
Hand grip strength right, kg	9	13.6 ± 6.9	15.6 ± 7.1	2.0	Paired *t*-test	0.057	0.74
Hand grip strength left, kg	9	13.4 ± 6.2	15.6 ± 6.9	2.1	Paired *t*-test	0.056	0.74
Six-minute walk test, m	14	407.6 ± 95.4	456.7 ± 83.1	49.1	Paired *t*-test	0.042	0.60

All analyses are based on participants with paired baseline and six-month observations for the respective outcome. Because the number of paired observations differed between outcomes, the paired sample size is shown separately for each row.

*P*-values are nominal and were not adjusted for multiple comparisons because all outcome analyses were exploratory.

Exploratory complete-case analyses showed nominal within-participant improvements in hand-eye coordination, reaction time, and six-minute walk distance. Hand-eye coordination improved from 67.5 ± 20.8 s at baseline to 56.7 ± 13.7 s at the six-month assessment (*n* = 15; nominal *p* = 0.002). Reaction time decreased from 0.372 ± 0.105 s to 0.332 ± 0.081 s (*n* = 15; nominal *p* = 0.002). Six-minute walk distance increased from 407.6 ± 95.4 m to 456.7 ± 83.1 m (*n* = 14; nominal *p* = 0.042).

No nominally significant within-participant changes were observed for static balance, flexibility, explosive strength, or sit-to-stand performance. Handgrip strength increased numerically on both sides but did not reach the nominal statistical significance threshold in the two-sided analysis.

### 3.6 Health-related quality of life

Paired PedsQL self-report data were available for 10 participants. (Table 4) Child-reported physical functioning increased from 56.0 ± 27.7 at baseline to 68.8 ± 26.3 at the six-month assessment (nominal *p* = 0.043). Child-reported emotional and social functioning also increased numerically, but these changes did not reach the nominal significance threshold.

**Table 4:** Changes in health-related quality of life between baseline and the six-month assessment.

PedsQL domain	Paired observations, n	Baseline T0, mean ± SD	Six months T1, mean ± SD	Mean change	Test	Nominal two-sided *p*-value	Effect size
Child self-report							
Physical functioning	10	56.0 ± 27.7	68.8 ± 26.3	12.8	Paired *t*-test	0.043	0.74
Emotional functioning	10	67.0 ± 27.5	77.0 ± 19.6	10.0	Paired *t*-test	0.255	0.38
Social functioning	10	78.0 ± 17.4	85.6 ± 14.3	7.6	Paired *t*-test	0.174	0.47
Parent proxy-report							
Physical functioning	13	49.9 ± 18.7	65.2 ± 24.1	15.3	Paired *t*-test	0.046	0.62
Emotional functioning	14	58.9 ± 21.3	68.2 ± 22.0	9.3	Wilcoxon	0.048	0.67
Social functioning	11	66.8 ± 20.2	80.5 ± 14.4	13.6	Paired *t*-test	0.007	1.02

Values are based on participants with paired observations for the respective domain. PedsQL scores range from 0 to 100, with higher scores indicating better health-related quality of life. Effect sizes are Cohen's dz for paired *t*-tests and matched-pairs rank-biserial correlation for the Wilcoxon signed-rank test. T1 denotes the six-month assessment.

*P*-values are nominal and were not adjusted for multiple comparisons because all outcome analyses were exploratory.

Parent proxy-report data were available for 11 to 14 participants, depending on the domain. Parent-reported physical functioning increased from 49.9 ± 18.7 to 65.2 ± 24.1 (nominal *p* = 0.046), emotional functioning from 58.9 ± 21.3 to 68.2 ± 22.0 (nominal *p* = 0.048), and social functioning from 66.8 ± 20.2 to 80.5 ± 14.4 (nominal *p* = 0.007).

Because these analyses were exploratory, based on small outcome-specific paired samples, and not adjusted for multiple testing, nominally significant within-participant changes were interpreted as potential outcome signals rather than as evidence of intervention efficacy.

### 3.7 Neuropsychological functioning

Paired neuropsychological data were available for 7 to 11 participants for the main cognitive domains and for 1 to 3 participants for the attentional subtests. Results are summarized in Table 5.

**Table 5:** Exploratory neuropsychological outcomes between baseline and the six-month assessment.

Domain	Paired observations, n	Baseline T0, mean ± SD	Six months T1, mean ± SD	Mean change	Test	Nominal two-sided *p*-value	Effect size
Main cognitive domains							
Fluid reasoning	10	50.5 ± 14.6	48.6 ± 7.6	−1.9	Paired *t*-test	0.676	−0.15
Verbal short-term memory	9	48.9 ± 11.8	44.1 ± 6.0	−4.8	Wilcoxon	0.953	−0.87
Verbal working memory	7	45.7 ± 9.3	46.2 ± 8.1	0.4	Paired *t*-test	0.896	0.05
Visual working memory	10	49.1 ± 8.1	52.7 ± 8.7	3.6	Paired *t*-test	0.330	0.34
Processing speed I	11	49.4 ± 9.6	52.0 ± 11.8	2.6	Paired *t*-test	0.236	0.39
Processing speed II	10	50.3 ± 9.6	54.1 ± 11.8	3.8	Paired *t*-test	0.201	0.45
Attentional subtests							
Intrinsic alertness	3	41.0 ± 5.6	43.0 ± 2.6	2.0	Descriptive only	—	—
Phasic alertness	3	40.0 ± 5.6	40.3 ± 5.5	0.3	Descriptive only	—	—
Inhibition, omissions	3	33.7 ± 12.7	38.0 ± 8.7	4.3	Descriptive only	—	—
Inhibition, errors	3	42.0 ± 18.4	51.7 ± 3.2	9.7	Descriptive only	—	—
Cognitive flexibility	1	50.0	61.0	11.0	Descriptive only	—	—
Cognitive flexibility, errors	1	39.0	66.0	27.0	Descriptive only	—	—

Values are age-standardized T-scores, with a normative mean of 50 and standard deviation of 10. Higher scores indicate better age-adjusted performance in the reported variables. Values are based on participants with paired observations for the respective outcome. Effect sizes are Cohen's dz for paired *t*-tests and matched-pairs rank-biserial correlations for Wilcoxon signed-rank tests. Formal inferential testing was not performed for attentional outcomes because paired sample sizes were extremely small. T1 denotes the six-month assessment.

*P*-values are nominal and were not adjusted for multiple comparisons because all outcome analyses were exploratory.

Fluid reasoning and verbal short-term memory showed small numerical decreases in age-standardized T-scores. Visual working memory and processing speed showed numerical increases. Processing speed I increased from 49.4 ± 9.6 to 52.0 ± 11.8, and processing speed II increased from 50.3 ± 9.6 to 54.1 ± 11.8. These changes did not reach nominal significance in the two-sided analyses.

Because paired data for the attentional subtests were available for only one to three participants, these outcomes were summarized descriptively without formal significance testing. The very small number of observations precludes meaningful interpretation of changes in attentional outcomes.

### 3.8 Participant and caregiver feedback

Feedback questionnaires were completed by 12 participants and 12 caregivers at the six-month assessment. Among responding participants, 6 of 11 (54.5%) reported that they enjoyed the exercise program, and 7 of 12 (58.3%) described the exercises as a welcome distraction from their illness. Among responding caregivers, 10 of 12 (83.3%) reported that their child's mood improved after exercise sessions.

Taken together, the feasibility indicators suggested that supervised individualized in-hospital exercise could be delivered repeatedly in routine care and was not associated with serious program-related adverse events. Uptake differed across program components: scheduled-session attendance was moderate using a conservative denominator, home-training documentation decreased over time, and SMART-Sport use was limited.

## 4 Discussion

This prospective single-center pilot study primarily evaluated the feasibility, uptake, safety, and acceptability of an individualized exercise program for children and adolescents undergoing cancer treatment. Exploratory analyses of motor performance, endurance, HRQoL, and neuropsychological functioning were included to describe potential outcomes signals and to inform future controlled studies, but the study was not designed to establish efficacy. Interpreted within this framework, the main finding is that supervised individualized in-hospital exercise could be implemented repeatedly in routine pediatric oncology care and was not associated with serious program-related adverse events. At the same time, feasibility was component-specific: scheduled-session attendance was moderate using a conservative denominator, documented home-based training decreased over time, and uptake of the optional SMART-Sport component was limited.

Because no predefined numerical feasibility threshold was specified, feasibility cannot be interpreted as having met a prespecified success criterion. Instead, feasibility was judged descriptively from the combined pattern of implementation indicators, including program delivery, scheduled-session attendance, reasons for non-attendance, safety, home-training documentation, SMART-Sport use, and participant and caregiver feedback. This combined interpretation supports feasibility most strongly for the supervised in-hospital component. The program was delivered by one sports therapist in routine care, 348 supervised sessions were attended, almost 60% of conservatively defined scheduled in-hospital opportunities resulted in participation, and no serious adverse event was attributed to the program. However, the absence of a predefined feasibility benchmark limits the strength of this conclusion and should be addressed prospectively in future studies.

The scheduled-session attendance rate requires careful interpretation. We deliberately used a broad denominator that included all scheduled exercise opportunities, even when attendance was prevented by absence from the hospital, reduced medical condition, concurrent treatment or diagnostic procedures, infection-control measures, or logistical reasons. This approach may underestimate willingness or ability to participate when exercise was medically and logistically possible. However, it reflects the practical challenge of implementing exercise support in routine pediatric oncology care, where treatment schedules, fluctuating health status, acute symptoms, and hospitalization patterns are integral parts of program delivery. Because participant presence and day-to-day medical suitability were not documented independently for all scheduled opportunities, a more restrictive attendance rate could not be calculated reliably. Thus, scheduled-session attendance should be understood as a conservative real-world indicator of implementation and uptake, not as adherence to a prescribed exercise dose.

The supervised in-hospital component appeared to be the most robust element of the program. Direct contact with the sports therapist allowed exercise content, intensity, and duration to be adapted immediately to each participant's daily condition, motivation, and treatment schedule. This flexibility is particularly important in pediatric oncology, where physical capacity may vary substantially from day to day. Comparable hospital-based or therapist-supervised interventions have reported favorable feasibility and participation, particularly when exercise was tailored to the child's clinical condition and embedded into the treatment setting (4, 14, 19, 24). The absence of serious program-related adverse events in the present study is also consistent with previous studies, systematic reviews, and consensus-based recommendations indicating that individualized exercise can be delivered safely during pediatric cancer treatment when adapted to medical status and supervised by appropriately trained staff (12–17, 21, 31, 42, 43). At the same time, this model required dedicated personnel, repeated scheduling efforts, and institutional support. Transferability to centers with fewer exercise-therapy resources may therefore be limited.

The home-based component was intended to extend exercise beyond the hospital setting and to allow families to continue individualized activities when participants were at home and medically able to exercise. Participants with available movement diaries documented a substantial number of home-training sessions, particularly during the first three months. However, documented home-training frequency decreased during the second half of the observation period, and diary data were missing for one quarter of the cohort. These findings suggest that home-based exercise may be feasible for some families but difficult to sustain during active pediatric cancer treatment. Treatment burden, fatigue, pain, nausea, hospitalization, fluctuating general condition, infection-control restrictions, and competing family demands may all have limited sustained participation. Age-dependent factors may also have contributed: younger children likely depended strongly on caregiver support, whereas adolescents may have required different motivational strategies and greater autonomy. Similar challenges in maintaining physical activity outside supervised settings have been describe previously (6, 20).

The home-training findings should be interpreted cautiously because movement-diary data were incomplete and reasons for missed home-training sessions were not systematically captured. Families who completed and returned diaries may have differed from those without diary data in motivation, caregiver availability, treatment burden, organizational capacity, or overall engagement with the program. Incomplete diary documentation may have underestimated actual home activity, whereas undocumented non-participation could not be distinguished from missing records. Future programs should therefore include structured follow-up contacts, simplified or digital documentation, and systematic recording of barriers to home participation. Age-specific strategies, caregiver support, and flexible adaptation to treatment phase and symptom burden may be necessary to sustain home-based exercise over longer periods.

The optional SMART-Sport component showed limited documented uptake and should be interpreted as regarded as a separate implementation finding rather than as a fully evaluated remote exercise intervention. Only four participants documented use of SMART-Sport between month 3 and the six-month assessment. Because the exact number of participants to whom SMART-Sport was offered was not systematically recorded and could not be reconstructed retrospectively, the uptake rate among eligible or offered participants could not be calculated. The finding therefore cannot be interpreted as a precise estimate of acceptability or uptake among suitable participants. Nevertheless, the limited documented use suggests that a passive optional video-based format may be insufficient to achieve sustained engagement during active pediatric cancer treatment. Delayed introduction, absence of fixed scheduling or real-time supervision, heterogeneous age and treatment burden, variable caregiver availability, motivational factors, and possible concerns about unsupervised exercise safety may all have contributed. Remote exercise components in pediatric oncology may therefore require more than access to standardized videos. Future approaches should evaluate scheduled live or hybrid sessions, regular therapist feedback, digital reminders, gamification, age-specific motivational strategies, and structured caregiver involvement (44, 45).

Exploratory complete-case analyses showed nominal within-participant improvements in selected motor-performance and endurance outcomes, including hand-eye coordination, reaction time, and six-minute walk distance. These findings are broadly consistent with previous exercise studies in pediatric oncology reporting improvements in motor performance, strength, functional capacity, or endurance following structured exercise interventions (16, 19, 24, 46). However, the present study lacked a control group, was underpowered, and analyzed multiple outcomes without adjustment for multiplicity. The observed changes may reflect the intervention, but they may also be explained by natural recovery, maturation, changes in treatment intensity, symptom relief, rehabilitation outside the study, repeated-test familiarity, regression toward the mean, or selection of participants who were well enough to complete follow-up assessments. Therefore, these results should be interpreted as hypothesis-generating outcome signals only.

The clinical relevance of the observed exploratory physical changes is difficult to determine. The approximately 49 m increase in six-minute walk distance may indicate improved functional endurance and appears potentially relevant at the individual level, particularly in children undergoing intensive treatment. However, no clinically meaningful change threshold was predefined, and established minimal clinically important differences for this heterogeneous pediatric oncology population during active treatment are not available. In addition, functional recovery over time may occur independently of the intervention, particularly when treatment intensity changes or acute symptoms improve (48) Future studies should prospectively define clinically meaningful endpoints and combine performance-based outcomes with patient- or caregiver-anchored assessments of perceived functional improvement.

HRQoL findings also require balanced interpretation. Child self-reports showed a nominal within-participant increase in physical functioning, whereas parent proxy-reports showed nominal increases in physical, emotional, and social functioning. These patterns may indicate that families perceived relevant improvements, particularly in physical functioning and psychosocial well-being. Prior studies and reviews have reported associations between physical activity, exercise interventions, fatigue reduction, and better HRQoL in children and adolescents with cancer or after cancer treatment (17, 23, 25, 47). However, HRQoL was assessed in small paired samples, and child self-reports and parent proxy-reports did not show identical patterns. Parent proxy-reports may reflect caregiver perceptions of the child's functioning and mood, while children and adolescents may evaluate their own symptoms, autonomy, and social participation differently. The caregiver-reported mood improvements after exercise sessions support the potential psychosocial value of direct, individualized exercise support, but participant-reported enjoyment was moderate and not uniform across the cohort. Thus, the HRQoL findings should be interpreted as potentially clinically relevant exploratory signals rather than as evidence of confirmed patient-centered benefit.

The neuropsychological findings were inconclusive and should not be overinterpreted. Paired sample sizes were small for the main cognitive domains and extremely limited for attentional measures, which were available for only one to three participants. Numerical changes were inconsistent, with small increases in some domains and little change or decreases in others. Although physical activity has been proposed to support neurocognitive functioning and some evidence suggests potential benefits in childhood cancer survivors, the available literature is heterogeneous with respect to population, timing, intervention type, and outcome measures (26–29). The present study cannot determine whether the exercise program influenced neuropsychological functioning. Larger studies with standardized neuropsychological endpoints, adequate paired sample sizes, and appropriate comparison groups are required to evaluate this question during active pediatric cancer treatment.

The study's contribution should therefore be viewed as preliminary and implementation-oriented. It does not provide controlled evidence of efficacy and does not establish that the exploratory outcome changes were caused by the intervention. Rather, it adds pragmatic data on the real-world delivery of individualized exercise support during active pediatric cancer treatment. In particular, it reports conservatively defined scheduled-session attendance, reasons for missed in-hospital sessions, home-training documentation over time, limited uptake of an optional video-based component, safety observations, and participant and caregiver feedback. These findings complement existing trials, systematic reviews, and guideline recommendations by highlighting practical barriers and component-specific differences in uptake during routine care.

Several limitations must be considered. First, the sample size was small, and only 15 of 20 participants completed at least one six-month follow-up assessment. The study was not powered to detect intervention effects, support subgroup analyses, or provide precise estimates of change. Second, the cohort was heterogeneous with respect to age, diagnosis, treatment modality, and treatment phase. This heterogeneity reflects real-world pediatric oncology care but limits interpretation of participation and exploratory outcomes in specific subgroups. Third, the study was uncontrolled and non-randomized; causal conclusions regarding efficacy are therefore not possible. Fourth, missing follow-up data, incomplete movement diaries, and outcome-specific complete-case analyses may have introduced attrition and selection bias. Participants with paired data may not represent the full enrolled cohort, and the direction of this bias cannot be determined.

Further limitations relate to the feasibility design and reporting. No predefined numerical feasibility threshold was specified, and feasibility was therefore interpreted descriptively rather than judged against a prespecified benchmark. The study was not registered prospectively in a public trials registry, and no formal sample-size calculation was performed. The omission of the three-month assessment from the present analysis precludes evaluation of the temporal pattern of change during the intervention period. SMART-Sport was optional, introduced only from month 3, and lacked a documented denominator of participants who were offered the component. The individualized nature of the intervention also limits reproducibility, although predefined exercise domains, institutional safety criteria, and standardized documentation were used to support consistency. Finally, the program was implemented at a single specialized center by one experienced sports therapist during the COVID-19 pandemic, which limits external validity and transferability to centers with different staffing, infrastructure, and patient-care routines.

From a practical perspective, the findings suggest that supervised individualized in-hospital exercise may be the component most readily implemented in specialized pediatric oncology care, provided dedicated staff and safety procedures are available. Home-based training may extend exercise support beyond the hospital but likely requires structured follow-up, caregiver involvement, and simplified documentation to maintain participation. Passive optional video-based formats appear insufficient as stand-alone remote exercise support and should be developed toward more interactive, scheduled, and personalized approaches. Future studies should be multicenter, prospectively registered, and designed with predefined feasibility criteria, clinically meaningful endpoints, standardized reporting of intervention delivery, and sufficient power to evaluate efficacy, sustainability, and scalability.

## 5 Conclusion

This prospective single-center pilot study supports the feasibility and safety of supervised individualized exercise support during pediatric cancer treatment under the conditions of a specialized center with a dedicated sports therapist. Feasibility was strongest for the supervised in-hospital component, whereas sustained home-based participation and uptake of the optional SMART-Sport component were limited. Exploratory outcome findings should be interpreted as hypothesis-generating only. Larger controlled studies with predefined feasibility criteria and clinically meaningful endpoints are needed to evaluate efficacy, sustainability, and scalability.

## Data Availability

The datasets generated and analyzed during the current study are not publicly available because they contain sensitive clinical data from a small cohort of pediatric oncology patients and may therefore compromise participant privacy. De-identified data supporting the conclusions of this article may be made available by the corresponding author upon reasonable request, subject to institutional regulations, applicable data protection requirements, and approval by the responsible ethics committee where required. Requests to access the datasets should be directed to michaela.kuhlen@uk-augsburg.de.
